# SPP1 overexpression is associated with poor outcomes in ALK fusion lung cancer patients without receiving targeted therapy

**DOI:** 10.1038/s41598-021-93484-2

**Published:** 2021-07-07

**Authors:** Xiaolin Ji, Yan Liu, Fang Mei, Xinyang Li, Mengxue Zhang, Buwen Yao, Rui Wu, Jiangfeng You, Fei Pei

**Affiliations:** Department of Pathology, Peking University Third Hospital, School of Basic Medical Sciences, Peking University Health Science Center, 38 Xue Yuan Road, Haidian District, Beijing, 100191 People’s Republic of China

**Keywords:** Non-small-cell lung cancer, Oncogenesis

## Abstract

The screening of non-small cell lung cancer (NSCLC) tumors for anaplastic lymphoma receptor tyrosine kinase (ALK) gene rearrangements is important because of the dramatically favorable therapy response to ALK inhibitor. However, the exact mechanism of poor survival in ALK fusion lung cancer patients without receiving targeted therapy is unclear. In this study, total of 521 tumor specimens from Chinese patients with lung cancer were screened for ALK fusion by immunohistochemistry (IHC) and confirmed by fluorescence in situ hybridization (FISH). As results, there were no cases of coexisting EGFR and ALK mutations identified. Fourteen cases (2.7%) harbored ALK fusion, including eight solid adenocarcinomas with signet ring cell features, four acinar adenocarcinomas with cribriform pattern containing mucin, one adenosquamous carcinoma and one micropapillary adenocarcinoma with mucin. Six (42.9%) of fourteen patients with ALK-positive lung cancer had stage IV disease, and five ALK-positive patients treated with platinum-based chemotherapy had poor outcome (all patients were dead and the mean survival time was 12 months), compared to 72 months for patients with ALK inhibitor therapy. Furthermore, Five ALK-positive cases were analyzed by whole exome sequencing (WES) and via direct transcript counting using a digital probe-base (NanoString) to explore the driver genes. Deregulation of PI3K/AKT signaling pathway in ALK-positive lung cancer was demonstrated by WES analysis, and significantly increased mRNA of ALK, ROS1, MET, SPP1 and PI3K signaling pathway was identified by NanoString assay. The concordance between NanoString, IHC and FISH methodologies for detecting ALK fusion was 100%. Significant overexpression of SPP1 protein in ALK-positive lung cancer was confirmed by IHC compared to paired adjacent normal tissues and ALK-negative cancers. Thus we concluded that SPP1 overexpression is associated with poor outcomes for patients with ALK fusion lung cancer without receiving targeted therapy and PI3K/AKT/SPP1 pathway may become the promising targets in patients with aggressive lung cancer.

## Introduction

Lung cancer is the second most common cancer and the leading cause of cancer-related deaths in the United States in 2019^1^. It is estimated that lung cancer accounts for 13% (228,150) of new cancer cases and 23–24% (142,670) of cancer deaths in 2019. In China, the morbidity and mortality of lung cancer is higher than the global average, there were 781,500 new lung cancer cases and 626,400 cancer death in China in 2014^2^, and non-small-cell lung cancer (NSCLC) accounts for 85% of all lung cancers^3^. However, NSCLC is often diagnosed at stage III and IV and has a poor prognosis before targeted therapy is available^4^. In recent genotyping efforts, approximately 50% of NSCLC were identified as having at least one oncogenic “driver”, with higher rates observed in younger and never smokers^5–8^. In a large Western patient population, screening identified 17.8% EGFR mutations, 27.6% KRAS mutations, and 4.4% ALK rearrangements^5^. TP53 (5%), PIK3CA (4%), CTNNB1(2%), BRAF (2%), NRAS (1%), HER2 (1%) and IDH (1% or less) mutations were also observed^6^. Furthermore, FGFR1 amplification (9.7–21%) and DDR2 mutations (2.2%) were discovered^7^. BRAF mutations (2–4.9%) have been shown to be associated with White race, female sex and ever-smokers. However, HER2 insertions (2.8%), RET rearrangement (1.2–1.9%), and ROS1 rearrangements (1.2–2.6%) were related to Asian race and never-smokers^7^. Inconsistently, spectrum of oncogenic driver mutations in lung adenocarcinomas from East Asian never-smokers was studied^8^. 75.3% harbored EGFR mutations, 6% had HER2 mutations, 5% had ALK fusions, 2% had KRAS mutations, and 1% harbored ROS1 fusions. Collectively, these findings suggested that most patients with NSCLC would benefit from targeted therapy.

Within these driver oncogenes, the anaplastic lymphoma kinase (ALK) gene rearrangement was identified in NSCLC in 2007^9^. ALK gene rearrangement is present in 3% to 5% of NSCLC and associated with unique clinical and pathologic features, including younger age, never or light smoking history, and adenocarcinoma histology^10^. Recognition of ALK-positive patients is clinically important because ALK rearrangements are associated with marked sensitivity to the ALK tyrosine kinase inhibitors (ALK-TKIs), such as Crizotinib, Ceritinib, Brigatinib, and Alectinib^4^. Patients who received genotype guided treatment had median overall survival of 4.25 years as compare to 2.4 years who did not received it^11^. Unfortunately, despite a favorable response to ALK-TKIs at initial treatment, patients eventually develop resistance to therapy (acquired resistance), and many ALK-positive patients do not respond to ALK-TKIs at all even in the initial therapy (intrinsic resistance)^12^.

However, the molecular mechanism of poor outcomes in ALK fusion lung cancer patients who did not receive targeted therapy is unclear. Early studies reported tumor or plasma levels of secreted phosphoprotein 1(SPP1, also known as osteopontin, OPN) were associated with an aggressive phenotype in lung cancer^13^. Secreted SPP1 interacts with integrin and CD44, which are associated with cellular transformation and tumor progression. Furthermore, SPP1 induces overexpression of urokinase-type plasminogen activator and increases cell migration, adhesion and metastasis^13^. Recently, increased SPP1 level was found in pediatric patients with ALK-positive anaplastic large cell lymphoma^14^. Therefore, we hypothesized that tumor levels of SPP1 would correlate with poor outcomes in patients with ALK fusion lung cancer who did not receive targeted therapy.

Here, we report the frequency of ALK fusion in a cohort of 521 Chinese lung cancer patients, and analyze the presence of ALK fusion in relation to clinicopathological characteristics and other driver genes mutations or expression (including SPP1) using whole exome sequencing (WES) and NanoString-based assay.

## Materials and methods

### Patients

We reviewed the clinical characteristics and histopathological specimens from 521 patients who were diagnosed as lung cancer and underwent surgical operation at Peking University Third Hospital, Beijing, China between September 2011 and September 2015, and the formalin-fixed, paraffin-embedded (FFPE) tumor materials are available for study. This study was approved by the Ethics Committee of the Peking University Health Science Center, and informed consent was obtained from all participants.

### Tumor pathology and EGFR mutation analysis

Tumor histology was classified according to World Health Organization criteria in 2015. DNA from formalin-fixed, paraffin-embedded (FFPE) tissue sections was prepared according to the manufacturer’s protocol (DNeasy Blood and Tissue Kit, Qiagen company). EGFR mutation analysis was conducted by amplification refractory mutation system (ARMS, AmoyDx company).

### Immunohistochemistry

Tumor specimens were fixed in 10% formalin overnight and embedded in paraffin. Sections of 4 μm thick were prepared for immunohistochemical staining. Endogenous peroxidase was quenched by freshly prepared 3% H_2_O_2_ with 0.1% sodium azide and then placed in antigen retrieval solution (0.01 mol/L citrate buffer, pH 6.0) for 15 min in a microwave oven at 100 °C and 600 W. Anti-ALK antibody (Cell signaling, clone D5F3, 1:100 dilution), anti-P53 (ZSGB-BIO,1:200 dilution)and anti-SPP1(ZSGB-BIO,1:200 dilution) was applied to the sections overnight at 4 °C, followed by incubation with second antibodies with horseradish peroxidase (Shanghai Gene Tech) for two hours. Then, the tissue sections were examined using DAB detection kit. Consistent negative control was obtained by replacement of primary antibody with PBS. SPP1 staining was scored as follows. Staining intensity was classified as 0 (negative), 1 (weak), 2 (moderate) and 3 (strong). Staining extent was dependent on the percentage of positive cells, which was divided into 0 (< 5%), 1 (5–25%), 2 (26–50%), 3 (51–75%) and 4 (> 75%). The final score was determined by multiplying the intensity and the quantity scores, which yielded a range from 0 to 12^13^.

We did not use positive control tissue for ALK on the IHC slides in our initial study. However, we added ALK(D5F3) staining of ganglion cells and axons in wall of appendix as the positive control (Figure S2).

### Fluorescence in situ hybridization

Fluorescence in situ hybridization of ALK (ALK FISH) was conducted on FFPE tissue using a dual-color break-apart probe specific to the ALK locus (Vysis LSI ALK Dual Color, Break Apart Rearrangement Probe; Abbott Molecular). Samples were considered positive if more than 15% of cells showed split ALK 5’ and 3’ probe signals or isolated 3’ signals^15^.

There were eight ALK(D5F3) IHC-positive but FISH-negative cases in our study, then, we selected 5 ALK IHC + /FISH + lung cancer samples and the corresponding pericarcinous (normal) tissues for the following WES and NanoString analysis. The five cases were not treated with ALK inhibitors.

### Whole exome sequencing

Five pairs of ALK-positive lung cancer samples were formalin fixed and paraffin embedded, and genomic DNA (gDNA) was extracted from ten 10-μm FFPE sections from matched tumor tissues and corresponding normal tissues using the QIAamp® Genomic DNA mini kit (Qiagen company) according to the manufacturer’s instructions. The DNA samples underwent further DNA quantification and qualification, genomic library preparation, clustering and sequencing, and bioinformatics analysis by Novogene (Tianjin, China). Briefly, a total of 0.6 μg of genomic DNA was used for sample preparation. Sequencing libraries were generated using an Agilent SureSelect Human All Exon kit V5 (Agilent Technologies, CA, USA). Finally, the DNA libraries were sequenced on Illumina Hiseq platform and 150 bp paired-end reads were generated. Valid sequencing data was mapped to the reference human genome (UCSC hg19) by Burrows-Wheeler Aligner (BWA) software to get the original mapping results stored in BAM format^16^. Samtools and bcftools were used to perform variant calling and identify single nucleotide polymorphisms (SNPs) and InDels^17^. Somatic SNVs were detected by MuTect^17^, somatic InDels were detected by Strelka^18^. SMGs (significantly mutated genes) that had obviously higher mutation rates than the background mutation rate were obtained by comprehensively analysing somatic SNVs and InDels. MuSiC software was used to perform convolution tests to identify SMGs^19,20^.

### Nanostring

For nCounter analysis, RNA was extracted from FFPE tissues with RNeasy FFPE Kit (Qiagen company) according to the manufacturer’s instructions. RNA concentration was estimated using the NanoDrop 2000 (Thermo Scientific). Total RNA was directly hybridized with a custom-designed multiplexed mixture of biotinylated capture tags and fluorescently labeled reporter probes located upstream (Elements Chemistry) complementary to target sequences of ALK, ROS1, RET, NTRK, BRAF and other driver genes (Table 1). The codeset also contained probes for housekeeping genes, positive and negative control probes were designed and synthesized by NanoString Technologies Inc. Briefly, 500 ng of total RNA was hybridized to nCounter probe sets for 16 h at 65℃. Samples were processed using an automated nCounter Sample Prep Station (NanoString Technologies, Inc., Seattle, WA). Cartridges containing immobilized and aligned reporter complex were subsequently imaged on an nCounter Digital Analyzer (NanoString Technologies, Inc.). Reporter counts were collected using NanoString’s nSolver analysis software version 4.0 (https://www.nanostring.com/products/analysis-software/nsolver)^21–23^.

**Table 1:** Target-specific oligonucleotide probes designed for nCounter assay.

Gene fusion probes (27 pairs)	ALK (8 variants)	ROS1 (6 variants)	RET (6 variants)	NTRK (5 variants)	BRAF (2 variants)
	EML4-ALK_V2	EZR-ROS1_E10-R34	CCDC6-RET_C1-R12	CD74-NTRK1__C7-N10	KIAA1549-BRAF_K16-B9
	EML4-ALK_V3a	GOPC-ROS1_G8-R35	KIF5B-RET_K15-R12	ETV6-NTRK3_E4-N15	KIAA1549_ex15 Fusion
	EML4-ALK_V5P	SDC43-ROS1_S2-R32	KIF5B-RET_K16-R12	ETV6-NTRK3_E5-N15	
	EML4_ex13 Fusion	SDC43_ex4 Fusion	KIF5B-RET_K22-R12	MPRIP-NTRK1_M21-N12	
	EML4_ex14 Fusion	SLC34A2-ROS1_S4-R32	KIF5B-RET_K23-R12	CD74_ex6 Fusion	
	EML4_ex2 Fusion	TPM3-ROS1	KIF5B4_ex24 Fusion		
	KIF5B-ALK_K17-A20				
	TFG-ALK				
Imbalance probes (37 pairs)	ALK(6 probes)	ROS1(6 probes)	RET(6 probes)	NTRK(13 probes)	BRAF(6 probes)
	ALK_ex14-18	ROS1	RET	NTRK1 _ex1-4	BRAF_ex13-14
	ALK_ex2-5	ROS1_ex18-24	RET_ex18-19	NTRK1 _ex15-16	BRAF_ex15-16
	ALK_ex22-24	ROS1_ex21-24	RET_ex19	NTRK1 _ex17	BRAF_ex18
	ALK_ex26-27	ROS1_ex39-40	RET_ex2-4	NTRK1 _ex6-8	BRAF_ex2-3
	ALK_ex29	ROS1_ex41-43	RET_ex4-5	NTRK1_ex13-14	BRAF_ex3-4
	ALK_ex8-12	ROS1_ex8-12	RET_ex6-9	NTRK1_ex4-5	BRAF_ex5-7
				NTRK2, NTRK3 _ex20	
				NTRK3 _ex10-13	
				NTRK3 _ex16-17	
				NTRK3 _ex18-19	
				NTRK3 _ex4-8, _ex8-9	
Other driver genes (62 pairs)	ABCB1, ABCG2, AKT1, AKT2, AXL, BCL2, BIRC5, BRCA1,CA9, CDK4,CDK6,CDKN2A,CTAG1B,EGFR, ERBB2,ERBB3,ERBB4,ERCC1, ESR1, FGFR1,FGFR2,FKBP1A,FLT1,FLT3,FLT4,FOLH1(PSMA),GRB7,GSTP1,HSP90AA1,HSP90AB1,IL2RA,IL6,IL8,JAK1,JAK2,KDR, KIT,MAP2K1,MAP2K2,MET,MTOR,PARP1,PDGFRA,PDGFRB,PGR,PIK3CA,PIK3CB,PIK3CD,PIK3CG,POSTN,RB1, RRM1, SLC29A1, SPINK1,SPP1(OPN),SRC,TIMP1,TOP1,TOP2A,TP53, TUBB3,TYMS				
Housekeeping genes	CLTC,GAPDH,GUSB,HPRT1,PGK1,TUBB (6 pairs)				
Positive control	POS_A,POS_B,POS_C,POS_D,POS_E,POS_F (6 pairs)				
Negative control	NEG_A,NEG_B,NEG_C,NEG_D,NEG_E,NEG_F (6 pairs)				

### Statistical analysis

Fisher exact test and Wilcoxon rank-sum test were used to assess the association of genotype status with clinicopathologic features, unless otherwise specified, using SPSS Statistics version 24.0. The data were subjected to student’s *t* test (two-tailed; a value of *p* < 0.05 was considered as statistical significance).

### Data availability statement

The data that support the findings of this study are openly available at http://doi.org/ or reference number and the data will be made available upon reasonable request.

### Statement for methods

All methods were carried out in accordance with relevant guidelines and regulations.

### Ethics approval and consent to participate

All studies concerning human tumor specimen were approved by the Ethics Committee of the Peking University Health Science Center, and informed consent was obtained from all patients.

## Results

### Screening patient characteristics

The clinical and pathologic features of 521 patients with lung cancer are summarized in Table 2. Only 4.2% (22/521) of stage IV disease underwent surgical resection of the primary tumor, and the patients were diagnosed with stage IV disease who were observed separate tumor nodules in a contralateral lobe or pleural tumor nodules during surgery without distant metastasis. Among the 521 patients, 173 cases had been assayed for the EGFR status by ARMS method, and mutation in EGFR was identified in 98 (56.7%) specimens. Furthermore, the total of 521 patients with lung cancer were screened for ALK fusion through immunohistochemistry of ALK (ALK IHC), and ALK FISH analysis was performed in ALK IHC positive cases. ALK rearrangements were found in 14 (2.7%) cases. The clinicopathologic characteristics of EGFR and ALK-positive patients with lung cancer are listed in Table 3. There were no cases of coexisting EGFR and ALK mutations identified. The ALK-positive patients were significantly younger than the patients harboring the EGFR mutations, with a median age of 55 compared with 63 (*P* < 0.001). However, there was no significant difference in sex, smoking history and histological pathology between ALK-positive and EGFR mutant patients. Furthermore, within the ALK-positive cohorts, 6 (42.9%) of 14 patients had stage IV disease, but only 10 (10.2%) of 98 patients with EGFR mutations had metastatic disease, which suggests a trend toward higher clinical stage among ALK-positive patients compared with EGFR mutant cohorts (*P* = 0.002).

**Table 2:** Clinicopathologic features of 521 patients with lung cancer.

Clinical characteristics	Total (n = 521)
**Age at diagnosis (years)**	
Median	62.43
Range	17–89
< 60 years	186 (35.7%)
≥ 60 years	335 (64.3%)
**Sex**	
Male	315 (60.5%)
Female	206 (39.5%)
**Pathology**	
Adenocarcinoma	323 (62.0%)
Squamous cell carcinoma	146 (28.0%)
Adenosquamous carcinoma	18 (3.5%)
Small cell carcinoma	18 (3.5%)
Large cell carcinoma	8 (1.5%)
Other lung cancer	8 (1.5%)

**Table 3:** The clinicopathologic characteristics of EGFR and ALK-positive patients with lung cancer.

Clinical characteristics	EGFR (n = 98)	ALK (n = 14)	*P*-value for ALK vs. EGFR
**Age at diagnosis (years)**			< 0.001
Median	63	55	
Range	28–79	17–74	
**Sex**			0.105
Male	44(44.9%)	6(42.9%)	
Female	54(55.1%)	8(57.1%)	
**Smoking history**^**a**^			0.235
Never	69(70.4%)	10(71.4%)	
Smoker	29(29.6%)	4(28.6%)	
**Pathology**			0.114
Adenocarcinoma	87(88.8%)	13(92.9%)	
Squamous cell carcinoma	5(5.1%)	0(0%)	
Adenosquamous carcinoma	4(4.1%)	1(7.1%)	
Large cell carcinoma	1(1.0%)	0(0%)	
Small cell carcinoma	1(1.0%)	0(0%)	
**Stage**^*b*^			0.002
Stage I	14(14.3%)	2(14.3%)	
Stage II	32(32.7%)	3(21.4%)	
Stage III	42(42.8%)	3(21.4%)	
Stage IV	10(10.2%)	6(42.9%)	

^a^Never smokers have smoked less than 100 cigarettes per lifetime. Smokers have smoked more than 100 cigarettes (current or former).

^b^Clinical stage represents stage at initial diagnosis. Stage was determined according to WHO TNM classification of lung carcinomas in 2015.

**Figure 1:**
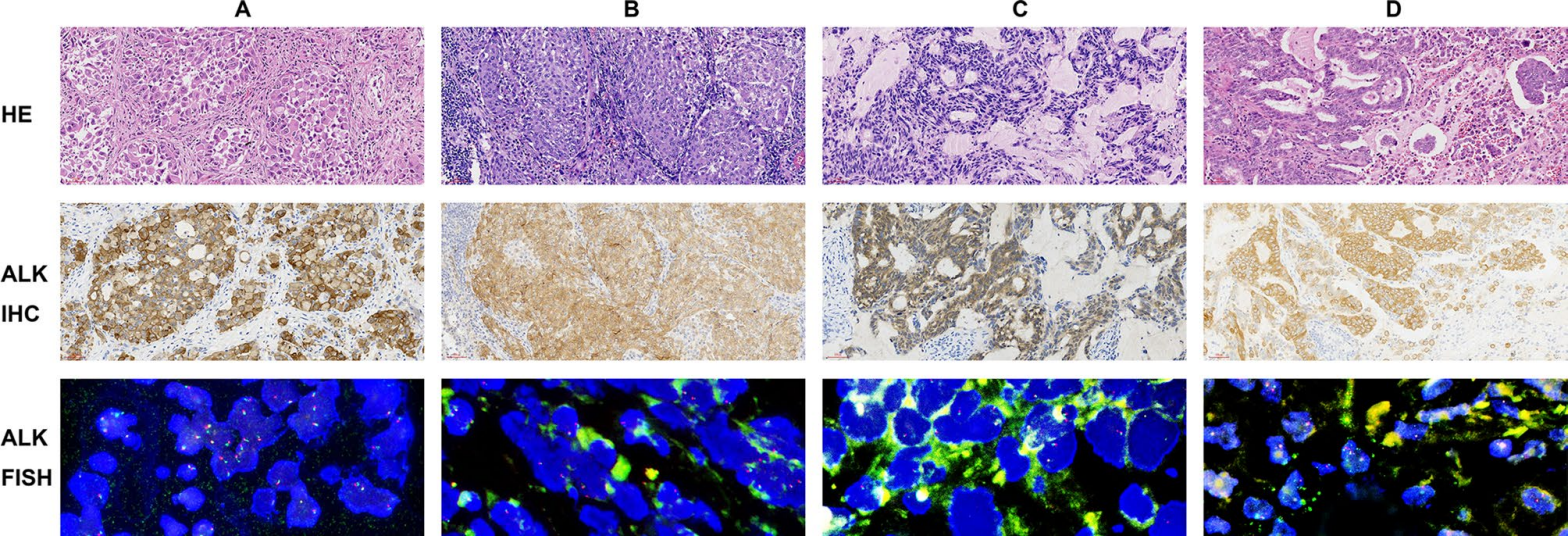
Histopathology of ALK-positive lung cancer and the corresponding ALK protein expression by IHC or ALK rearrangement by FISH. (**A**) Solid adencarcinoma with signet ring cell features. (**B**) Adenosquamous carcinoma. (**C**) Acinar (cribriform pattern with mucin) adenocarcinoma. (**D**) Micropapillary adenocarcinoma with mucin. Scale bars: 50 µm (magnification of HE and IHC, × 200).

**Figure 2:**
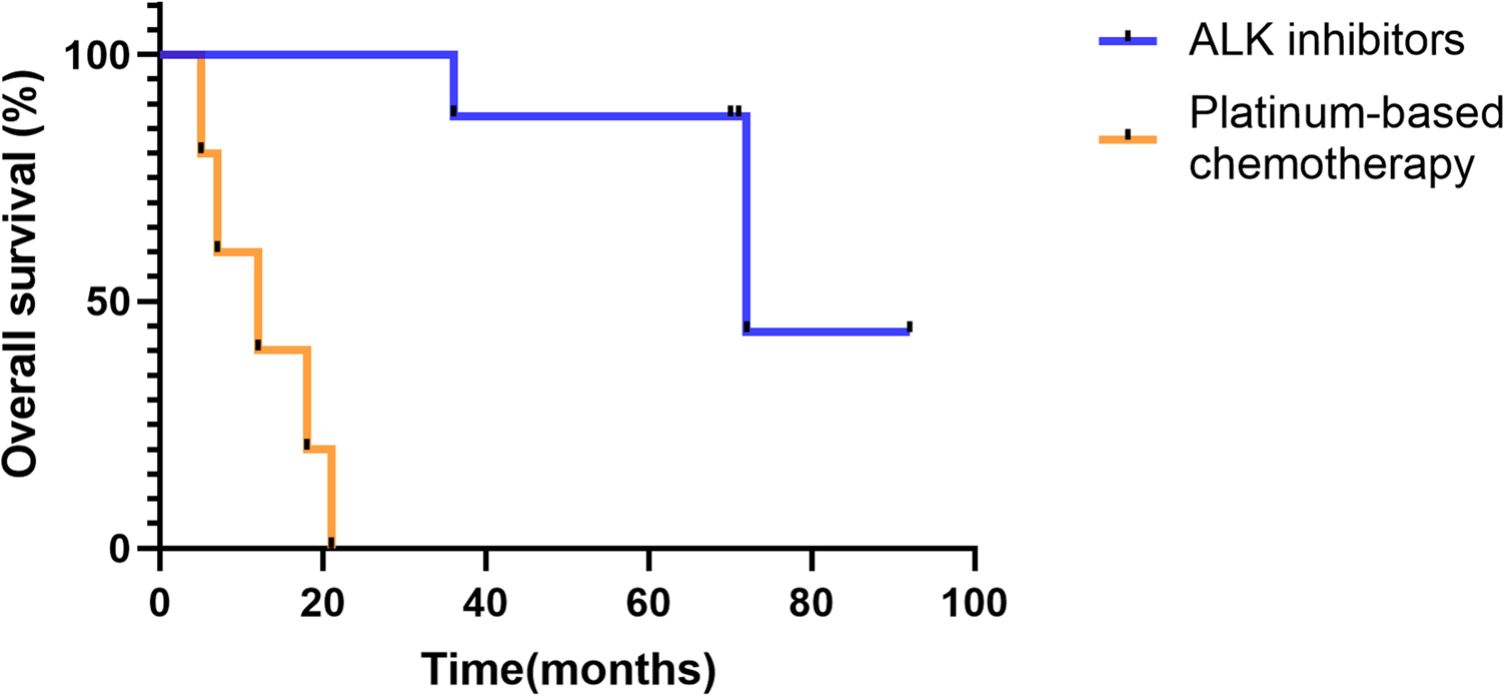
Overall survival (OS) of patients with ALK-positive lung cancer receiving ALK inhibitors or platinum-based chemotherapy.

The clinicopathologic findings of 14 patients with ALK rearrangement are summarized in Table 4. Of 14 patients with ALK-positive lung cancer, 8 (57.1%) were diagnosed histopathologically as solid adenocarcinoma with signet ring cell features, 2 (14.1%) were diagnosed as mixed solid (with signet ring cell) and acinar (cribriform pattern with mucin) adenocarcinoma, 1 (7.2%) was diagnosed as adenosquamous carcinoma, 1 (7.2%) was diagnosed as mixed papillary, solid (with signet ring cell) and acinar (cribriform pattern with mucin) adenocarcinoma, 1 (7.2%) was diagnosed as mixed solid (with signet ring cell) and acinar adenocarcinoma, and 1 (7.2%) was dignosed as micropapillary adenocarcinoma with mucin (Fig. 1). Furthermore, we estimated clinical outcome for ALK-positive patients with lung cancer treated with ALK inhibitor or platinum-based chemotherapy. 6 patients (42.9%) with ALK-positive lung cancer were still alive, 7 patients (50%) had died, and one patient (7.1%) had been lost to follow-up. For ALK-positive patients treated with ALK inhibitors, the median overall survival (OS) was 72 M, compared with 12 M for patients treated with platinum-based chemotherapy (*P* = 0.0001, Fig. 2). Thus, ALK inhibitors have demonstrated superior efficacy to platinum-based chemotherapy as front-line treatment for patients with ALK-positive lung cancer.

**Table 4:** The clinicopathologic findings of 14 patients with ALK rearrangement.

Patient No.	Sex	Age	Smoking status	Histology	Stage^a^	Survival	ALK inhibitors use
1	Female	74	No	Solid adenocarcinoma, signet	IV	Dead,18 M	No
2	Male	33	Yes	Adenosquamous carcinoma	IIa	Dead,21 M	No
3	Male	52	Yes	Solid adenocarcimona, signet	IIIb	Dead,12 M	No
4	Female	69	No	Papillary,solid(signet),cribriform pattern of acinar with mucin	IV	Alive,7Y8M	Yes
5	Female	66	No	Solid adenocarcinoma, signet	IV	Dead,5 M	No
6	Female	56	No	Solid adenocarcinoma, signet	IV	Dead,7 M	No
7	Male	39	Yes	Solid(signet), acinar	IV	Dead, 6Y	Yes
8	Male	58	No	Cribriform pattern of acinar with mucin, solid adenocarcinoma	II	Alive, 5Y11M	Yes
9	Female	67	No	Solid adenocarcinoma, signet	II	Alive, 5Y10M	Yes
10	Female	69	No	Solid adenocarcinoma, signet	III	Unknown	No
11	Female	60	No	Solid(signet), cribriform pattern of acinar with mucin	IV	Alive, 5Y10M	Yes
12	Male	17	No	Solid adenocarcinoma, signet	IV	Dead, 3Y	Yes
13	Male	53	Yes	Solid adenocarcinoma, signet	Ia	Alive, 5Y10M	Yes
14	Male	61	No	Micropapillary with mucin	Ia	Alive, 5Y10M	Yes

^a^Clinical stage represents stage at initial diagnosis. Stage was determined according to WHO TNM classification of lung carcinomas in 2015.

### Deregulation of PI3K/AKT signaling pathway in ALK-positive lung cancer using WES analysis

**Figure 3:**
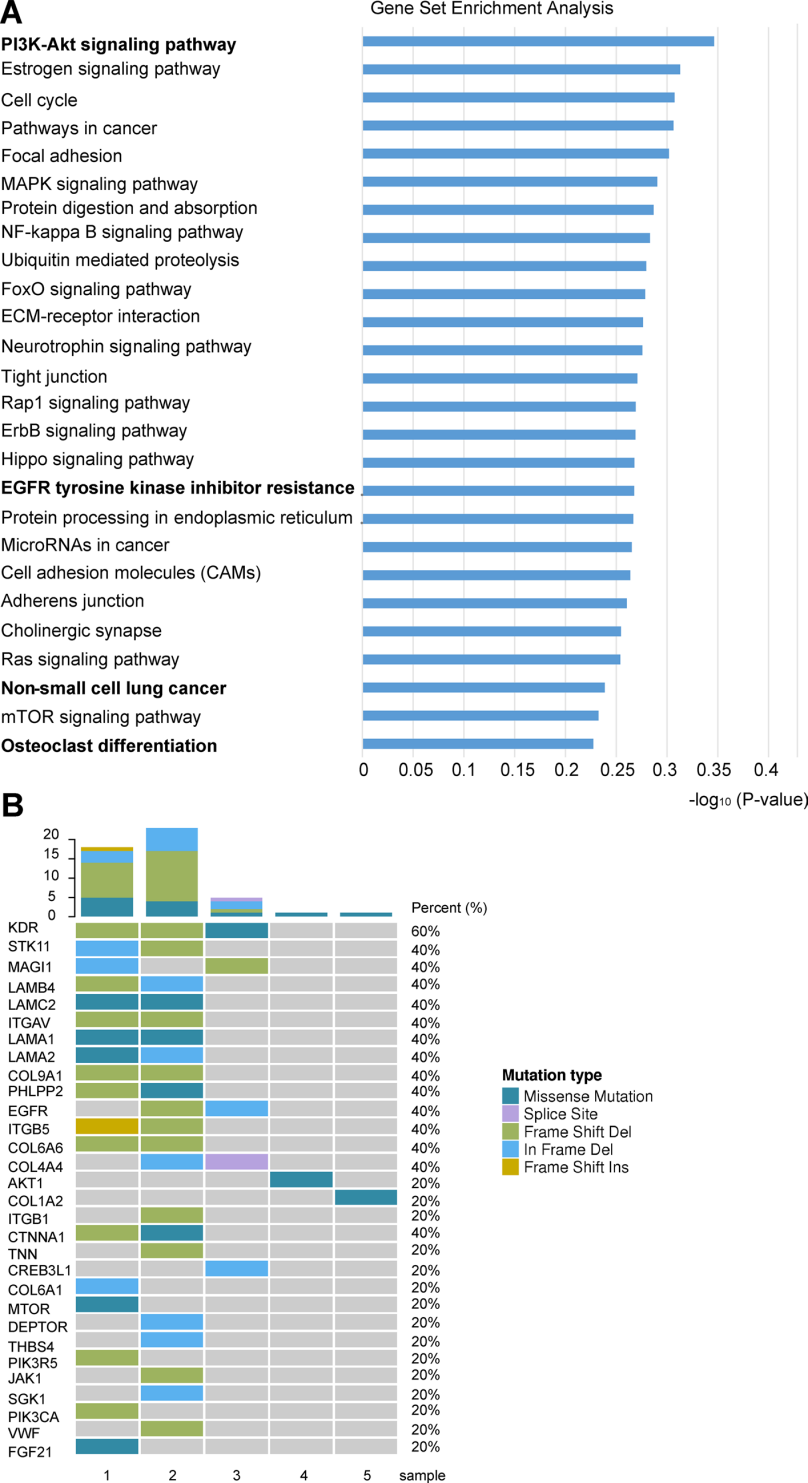
Analysis of significantly mutated genes (SMGs) revealing PI3K/AKT signaling pathway deregulation in 5 ALK-positive lung cancer samples compared to paired adjacent normal tissues through whole-exome sequencing. (**A**) Pathway enrichment of SMGs. The bar graph shows that KEGG passway terms are enriched in SMGs, the X axis indicates *P* values in the specified formula. All KEGG terms are statically significant (adjusted *P* value < 0.05). (**B**) Landscape of significantly mutated genes related to PI3K/AKT signaling pathway.

To identify significantly mutated genes (SMGs) and pathways involved in ALK-positive lung cancer, WES analysis was performed on 5 ALK-positive lung cancer samples and the corresponding pericarcinous (normal) tissues. Interestingly, we demonstrated that PI3K/AKT signaling pathway, EGFR tyrosine kinase inhibitor resistance, non-small cell lung cancer and osteoclast differentiation were associated with ALK-positive lung cancer (Fig. 3A), all KEGG terms are statically significant (adjusted *P* value < 0.05). Moreover, KEGG enrichment analysis revealed that 30 genes were enriched in PI3K/AKT signaling pathway (Fig. 3B).

In the five ALK-positive cases analyzed by whole exome sequencing (WES), only one case with EML4-ALK-V3a fusion diagnosed as mixed acinar (cribriform pattern with mucin) and solid adenocarcinoma carried TP53 mutation(exon 4; c.A416T; p.E139V). The finding is listed in Table S1.

### Increased mRNA of ALK, ROS1, MET, SPP1 and PI3K signaling pathway in ALK-positive lung cancer identified by NanoString assay

**Figure 4:**
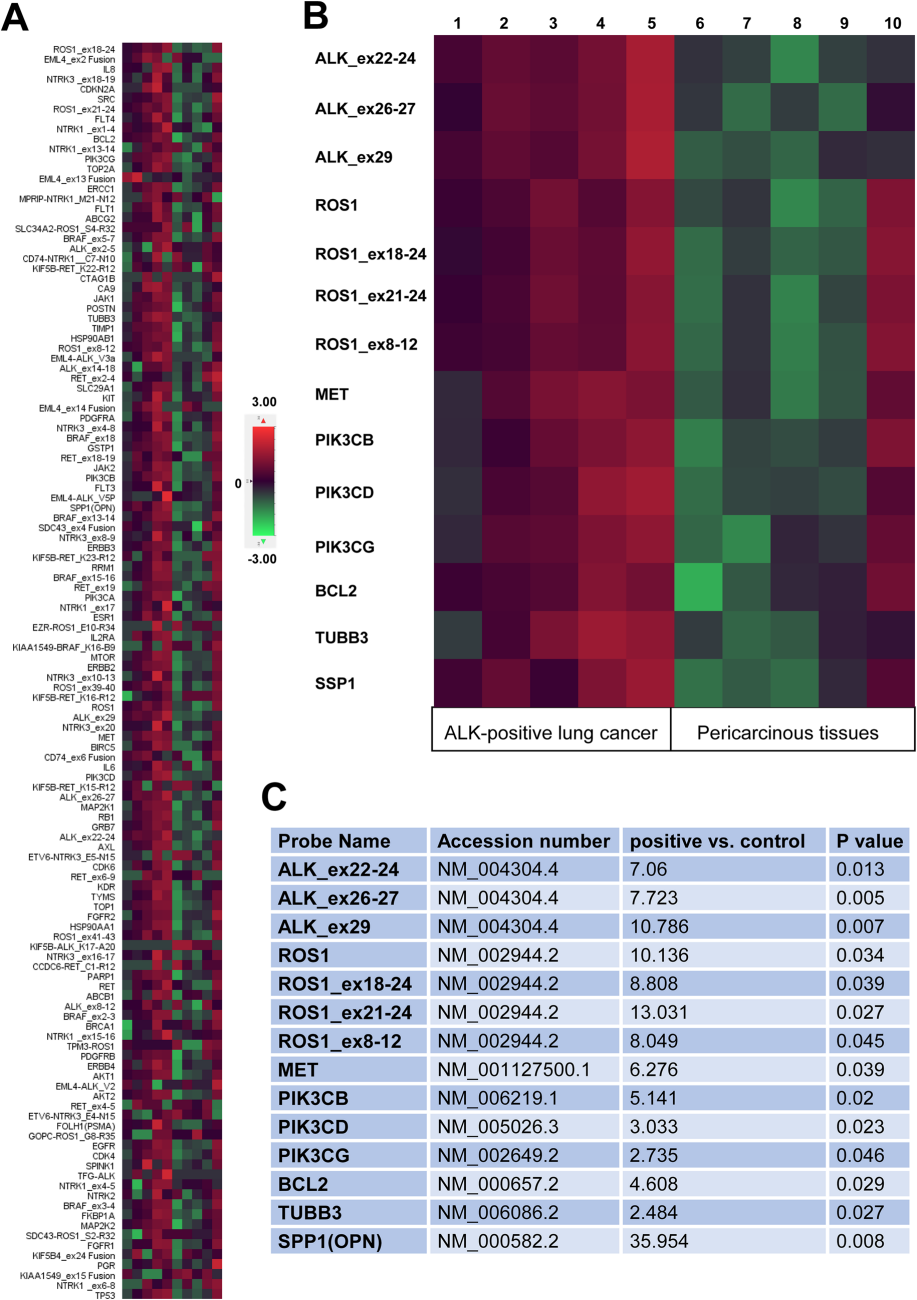
Increased mRNA of ROS1, MET, SPP1 and PI3K signaling pathway in ALK-positive lung cancer identified by NanoString assay. (**A**) Heatmap showed the mRNA expression of 67 genes in 5 ALK-positive lung cancer samples and the corresponding pericarcinous (normal) tissues. (**B**) Heatmap revealed significant mRNA overexpression of ALK (exon 22–24, exon26-27, and exon 29), ROS1, MET, PIK3CB, PIK3CD, PIK3CG, BCL2, TUBB3 and SPP1, respectively (*P* < 0.05). (**C**) Significant differences of gene counts between 5 ALK-positive lung cancer cases and the corresponding pericarcinous tissues (*P* < 0.05). Heatmap was created using NanoString’s nSolver analysis software version 4.0 (https://www.nanostring.com/products/analysis-software/nsolver).

We further analyzed mRNA expression of 67 genes in the same 5 ALK-positive lung cancer samples and the corresponding pericarcinous (normal) tissues by NanoString assay. The expression of ALK mRNA (exon 22–24, exon26-27, and exon 29) is shown in Fig. 4 and Table 5, respectively. The concordance between the NanoString, immunohistochemistry and FISH methodologies was 100%.

**Table 5:** Gene counts of 5 ALK-positive lung cancer cases and the corresponding pericarcinous tissues by NanoString assay.

Probe name	Case 1	Case 2	Case 3	Case 4	Case 5	Control 1	Control 2	Control 3	Control 4	Control 5
	Solid (signet)	Solid (signet)	Micropapillary with mucin	Cribriform pattern of acinar with mucin, solid	Papillary,solid(signet),cribriform pattern of acinar with mucin					
ALK_ex22-24	39	86	66	137	587	7	5	1	5	7
ALK_ex26-27	23	119	88	153	757	7	2	6	2	18
ALK_ex29	64	127	92	217	913	6	8	5	17	15
EML4-ALK_V3a	3	14	70	269	99	4	4	4	2	18
EML4-ALK_V5P	2	1	1	3	1035	5	3	2	1	16
EML4_ex13 Fusion	59	180	2	4	3	6	1	3	3	4
ROS1	88	172	766	234	1544	7	13	1	3	1276
ROS1_ex18-24	60	128	472	313	1858	4	14	5	7	1296
ROS1_ex21-24	91	194	976	438	2554	2	15	1	8	1672
ROS1_ex8-12	81	107	359	270	1513	2	11	1	4	1226
MET	19	172	603	975	638	5	16	2	6	273
PIK3CB	23	62	176	300	483	4	13	11	15	330
PIK3CD	15	56	74	274	384	5	10	11	9	70
PIK3CG	14	75	88	197	306	4	2	15	11	107
BCL2	45	62	82	269	175	1	6	16	19	169
TUBB3	8	31	93	187	141	8	4	5	13	16
SPP1(OPN)	466	2082	349	6292	20,921	5	11	6	62	1056

Among the 5 ALK-positive cases with lung cancer, the following variants were identified (Fig. 4 and Table 5): three (60%) exhibited EML4-ALK-V3a fusion (cases 3, 4 and 5), one (20%) exhibited EML4-ALK-V5p fusion (case 5) and two (40%) exhibited EML4_ex13 fusion (cases 1 and 2). The histopathological findings of the three cases with EML4-ALK-V3a fusion are micropapillary adenocarcinoma with mucin, mixed solid (with signet ring cell) and acinar (cribriform pattern with mucin) adenocarcinoma and mixed papillary, solid (with signet ring cell) and acinar (cribriform pattern with mucin) adenocarcinoma, respectively. The histopathological finding of the one case with EML4-ALK-V5p fusion is mixed papillary, solid (with signet ring cell) and acinar (cribriform pattern with mucin) adenocarcinoma. The histopathological findings of the two cases with EML4_ex13 fusion are solid adenocarcinoma with signet ring cell features.

Furthermore, we found mRNA overexpression of ROS1 (10.136-fold), ROS1_ex18-24 (8.808-fold), ROS1_ex21-24 (13.031) and ROS1_ex8-12 (8.049-fold) in the ALK-positive lung cancer samples compared to the corresponding pericarcinous tissues (*P* < 0.05), especially cases 3, 4 and 5. We also found the level of MET mRNA increased in the five cases (*P* = 0.039), especially cases 2, 3, 4 and 5. Excitingly, the five cases with ALK rearrangements also exhibited significant mRNA overexpression of PIK3CB (5.141-fold), PIK3CD (3.033-fold), PIK3CG (2.735-fold), BCL2 (4.608-fold), TUBB3 (2.484-fold) and SPP1 (35.954-fold), respectively (*P* < 0.05).

Then, we further analyzed mRNA expression of TP53 in the same five ALK-positive lung cancer samples and the corresponding pericarcinous (normal) tissues by NanoString assay, and we found mRNA overexpression of TP53 in the ALK/TP53 co-mutated lung cancer sample (case 4) compared to the corresponding pericarcinous tissues (45-fold). The mRNA expression of TP53 by NanoString assay is listed in Table S2.

### Overexpression of SPP1 protein in ALK-positive lung cancer confirmed by immunohistochemistry

**Figure 5:**
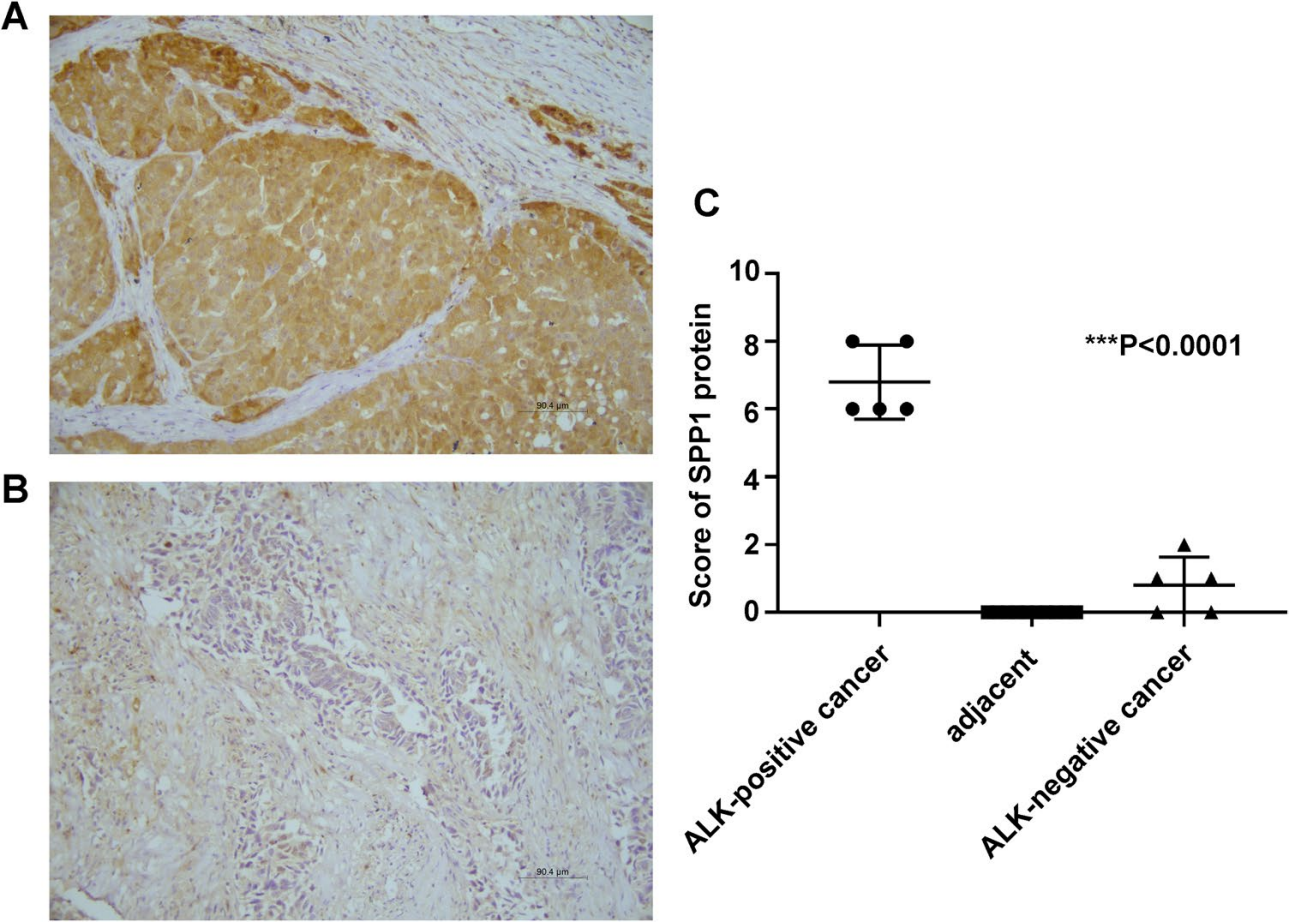
Immunohistochemical staining of SPP1. (**A**) ALK-positive lung cancer. (**B**) ALK-negative lung cancer. Magnification, × 200, scale bars: 90.4 µm. (**C**) The scores were determined by evaluating the percentage and intensity of immunopositivity and were analyzed by paired-samples t test. There was a significant upregulation of SPP1 expression in ALK-positive lung cancers compared to ALK-negative lung cancers and paired adjacent normal tissues (**P < 0.0001).

To further confirm expression of SPP1 protein in ALK fusion lung cancer, 5 ALK-positive and 5 ALK-negative lung cancer samples with paired adjacent normal tissues were collected. Immunohistochemical staining showed that SPP1 staining pattern in lung cancer was cytoplasmic, and the average scores for 5 ALK-positive and 5 ALK-negative lung cancer samples were 6.8 ± 1.095 and 0.8 ± 0.837, respectively. Therefore, we found a significant upregulation of SPP1 expression in ALK-positive lung cancers compared to ALK-negative lung cancers and paired adjacent normal tissues (*P* < 0.0001, Fig. 5).

Finally, we confirmed overexpression of P53 protein in the ALK/TP53 co-mutated lung cancer sample (case 4) compared to the only ALK-rearranged cases by immunohistochemistry, and the latter expressed only a small amount of wild-type P53 protein (Figure S1).

## Discussion

In the current study, our data revealed 98 (56.7%) EGFR mutations in 173 patients with lung cancer and 14 (2.7%) ALK fusions in 521 patients. The 14 ALK-positive cases were confirmed by both IHC and FISH, however, there were eight ALK IHC-positive but FISH-negative cases which were not identified as ALK fusions in our study. It was similar to previous studies. ALK fusion oncogene is reported in 2–7% of advanced lung cancers^24^. Five (6.7%) out of 75 Japanese NSCLC patients were positive for EML4-ALK fusion from initial reports of Soda^9^. A rate of 3.6% was reported in Korean patients, while a frequency of 6.6% for ALK fusion was described in 1,504 Chinese Han patients^25^. A lower frequency of 1.4% was reported in 136 samples obtained from Caucasian patients^24^. Moreover, there were no cases of coexisting EGFR and ALK mutations identified. The result was consistent with previous reports. It was reported that no cases of coexisting EGFR mutations and ALK rearrangements across 1,687 Western patients with NSCLC^5^. Besides, patients with ALK-positive lung cancers were significantly younger and were found at higher clinical stage at diagnosis compared with EGFR mutant cohorts. The difference in median age between ALK-positive patients and patients with EGFR mutation exceeded 8 years. Interestingly, several other tumors known to harbor ALK fusions, such as anaplastic large cell lymphomas, neuroblastomas, and inflammatory myofibroblastic tumors, are also associated with younger age and are most common in children and young adults^10^. Our data also described 42.9% of patients with ALK-positive lung cancer was found at stage IV at diagnosis and the median overall survival was only 12 M in patients who did not receive targeted therapy, this finding is consistent with previous reports. Patients with ALK-positive lung cancer appear to have a worse prognosis than those without ALK fusion^25^. ALK translocation has been associated with an increased risk of brain involvement, liver metastases and a greater number of metastatic sites. However, the molecular mechanism of poor outcomes in ALK fusion lung cancer patients who did not receive targeted therapy is unclear.

To further explore the molecular mechanism of poor outcomes in patients with ALK fusion lung cancer who did not receive targeted therapy, we have identified significantly mutated genes (SMGs) and pathways on 5 ALK-positive lung cancer samples and the corresponding pericarcinous (normal) tissues using whole exome sequencing (WES) analysis. Our finding revealed deregulation of PI3K/AKT signaling pathway, EGFR tyrosine kinase inhibitor resistance, non-small cell lung cancer and osteoclast differentiaton in ALK-positive lung cancer. Similar observations were made by other investigators. ALK fusions have clear oncogenic potential as its aberrant tyrosine kinase activity enhances cell proliferation, survival and leads to cytoskeletal rearrangements or change in cell shape^26^. Moreover, ALK fusions activate many different pathways, including the phosphatidylinositol 3-kinase (PI3K)-Akt pathway. ALK fusions binds to and activates PI3K through the regulatory p85 subunit of PI3K in tumor cells, leading to the phosphorylation of its downstream effectors AKT1 and AKT2^26^. Combined TAE684 (ALK tyrosine kinase inhibitors) with PI3K inhibitor synergistically inhibited the proliferation of EML4-ALK-positive lung cancer cells and inhibition of PI3K/AKT signaling may conquer resistance to ALK-targeted treatment^27^. Suprisingly, we firstly reported osteoclast differentiation in ALK-positive lung cancer in the world. This puzzling result may not easily be explained. Nonetheless, a case of inflammatory myofibroblastic tumor with ALK rearrangement displayed unusual histologic features of high cellularity with a herringbone pattern and tumor-associated osteoclast-like giant cells and suggested a trend toward more aggressive behavior^28^.

We further analyzed mRNA expression of 67 genes in 5 ALK-positive lung cancer samples and the corresponding pericarcinous (normal) tissues by NanoString assay, and found increased mRNA of ALK, ROS1, MET, SPP1 and PI3K signaling pathway in ALK-positive lung cancer. In the present study, we sought to implement the NanoString panel of ALK fusion detection for lung cancer patients, our NanoString results were further compared with immunohistochemistry or FISH analysis of ALK and showed a full concordance of methodologies. Of the five ALK-positive cases, three (60%) exhibited EML4-ALK-V3a (cases 3, 4 and 5), one (20%) exhibited EML4-ALK-V5p (case 5) and two (40%) exhibited EML4_ex13 fusion (cases 1 and 2). These results are in agreement with previous studies^29^. Among the methodologies currently available for ALK fusion detection, only NanoString and next-generation sequencing (NGS) can detect different variants of ALK rearrangements which exhibit distinct sensitivity to Crizotinib. NGS was especially useful for detecting ALK rearrangements, including those with new fusion partners, but it has some disadvantages, such as the higher cost, and it requires more effort for analysis^30^. However, NanoString provides an interesting option because of its hands-on time, quantitative precision, reproductive results in low quality and quantity RNA from FFPE (since it is performed without amplification and sequence errors)^21^. Nonetheless, NanoString has some limitations because it is impossible to detect novel fusion partners.

Interestingly, we found increased mRNA of ROS1 and MET in ALK-positive lung cancer, the molecular mechanism is unclear at present. Recently, kinase inhibitor such as Crizotinib for ALK fusions has shown therapeutic efficacy in NSCLC patients carrying other rearrangements, including ROS1 (c-ros oncogene 1), RET (rearranged during transfection), MET amplification and MET exon 14 deletion^22^. This maybe partly explain our results. Some finding revealed that positivity for ALK, ROS1 and RET was mutually exclusive^23^, However, a few cases of concurrent ALK and ROS1 rearrangements had been reported recently^31^. Concurrent genetic alterations were detected in 5.4% of lung adenocarcinoma patients, and EGFR mutations were observed as the most common partner for concurrent genetic alteration^32^. Significantly more concurrent genetic alterations were observed in older patients. For example, a 77-year-old never-smoker woman with lung adenocarcinoma was reported to be refractory to Gefitinib and responsive to Crizotinib with concurrent rare mutation of EGFR (L861Q) and increased ALK/MET/ROS1 gene copy number^33^.

Furthermore, we found increased mRNA of PIK3CB, PIK3CD, PIK3CG, BCL2, TUBB3 in ALK-positive lung cancer, which suggested activation of PI3K/AKT signaling pathway. The findings were in agreement with our WES results. PI3K pathway alterations are found in 11% of lung adenocarcinoma^34^, and activated PI3K induces the phosphorylation and activation of AKT, which in turn phosphorylates multiple targets to control cell proliferation^35^. PI3K are composed of an 110-KD catalytic subunit and an 85-KD adaptor subunit. In mammals, there exist four isoforms of PI3K catalytic p110 subunits: p110α(PIK3CA), p110β(PIK3CB), p110γ (PIK3CG), and p110δ (PIK3CD)^36^. PIK3CG and PIK3CD isoforms are mainly restricted to functions in immune cells where they are expressed, whereas PIK3CA and PIK3CB are ubiquitous in most tissues and associated with many tumors^37^. Overexpression of either PIK3CB, PIK3CG or PIK3CD is sufficient to transform chicken embryo fibroblasts, suggesting an inherent oncogenic potential of these proteins^38^. Moreover, BCL2 is one of PI3K/AKT pathway apoptotic related proteins^39^. TUBB3 (class III β-tubulin) is a microtubule protein that generates highly dynamic microtubules^40^. TUBB3 is related to a poor prognosis and the metastasis of many malignancies. In lung cancer, TUBB3 promotes tumorigenesis, EMT, and anoikis resistance through the PI3K/AKT pathway. TUBB3 plays a crucial role in the resistance to EGFR-TKI in patients with NSCLC and is associated with the activation of PI3K/AKT signaling.

Excitingly, our finding revealed a dramatical increase of SPP1 mRNA (35.954-fold) in ALK-positive lung cancers using NanoString analysis and then overexpression of SPP1 protein was confirmed by IHC. The obvious increase of SPP1 mRNA and protein in ALK-positive lung cancers is attracting considerable interest. SPP1 is a member of small integrin-binding ligand N-linked glycoprotein family of chemokine-like calcified extracellular matrix-associated glyco-phosphosialo protein, initially discovered in transformed, malignant epithelial cells^41^. SPP1 sustains angiogenesis via activation of PI3K/AKT pathway. Moreover, SPP1 plays a crucial role in EMT and SPP1 overexpression levels have also been associated with metastasis of lung cancers, colorectal cancers and melanomas. There is a significant reverse correlation between SPP1 expression and overall survival in lung cancer^42,43^. They observed a statistically significant relation between high expression of SPP1 (osteopontin) and shorter overall survival and disease-free survival even in patients with stage I lung cancers^43^. SPP1 binds to integrins and CD44, and then contains a protease-hypersensitive site. SPP1 interacts with integrins to induce the activation of PI3K/AKT pathway in cancer cells. The SPP1-CD44 interaction contributes to the survival of cancer cells by activating PI3K/AKT signaling^42^. Recent reports have indicated that SPP1 activity is regulated by HIF1α through PI3K/AKT pathway-dependent manner. Our study demonstrated that abnormally high levels of SPP1 were present in patients with ALK-positive lung cancers, similar to previous findings in pediatric patients with ALK-positive anaplastic large cell^14^. Lung cancers show a predilection to metastasize to bone, and the number of osteoclasts is increased at metastatic sites, which is accordance with osteoclast differentiation of ALK-positive lung cancer cells in our findings^41^. We hypothesized that ALK-positive lung cancer cells augment the activity of bone resorption via promoting the differentiation of osteoclasts by secreting SPP1 and lead to osteolytic bone metastasis. For the first time, we revealed that SPP1 overexpression is associated with poor outcomes in ALK fusion lung cancer patients who did not receive targeted therapy. Thus, targeting SPP1 may be an attractive therapeutic approach for aggressive lung cancer.

## Supplementary Information

Supplementary Legends.

Supplementary Fig. S1.

Supplementary Fig. S2.

Supplementary Table S1.

Supplementary Table S2.

## Data Availability

The datasets used and/or analyzed during the current study are available from the corresponding author upon reasonable request.
