# Supplementary material for: SPP1 overexpression is associated with poor outcomes in ALK fusion lung cancer patients without receiving targeted therapy
Source: Sci Rep. 2021 Jul 7;11:14031. doi: 10.1038/s41598-021-93484-2 (PMC8263595; doi:10.1038/s41598-021-93484-2)
Supplement: Supplementary file 1 — Supplementary Legends. [file 41598_2021_93484_MOESM1_ESM.docx]

**Supplemental Figures and legends**

**Figure S1: Immunohistochemical staining of P53. A,** ALK/TP53 co-mutated lung cancer sample (case 4). **B,** The only ALK-rearranged cases. Magnification, 400, scale bars: 20 µm.

**Figure 2: We used ALK(D5F3) staining of ganglion cells and axons in wall of appendix as the positive control. A,** HE staining. **B,** ALK(D5F3) staining. Magnification, 200, scale bars: 100 µm.
