# Supplementary figures and images for: SPP1 overexpression is associated with poor outcomes in ALK fusion lung cancer patients without receiving targeted therapy
Source: Sci Rep. 2021 Jul 7;11:14031. doi: 10.1038/s41598-021-93484-2 (PMC8263595; doi:10.1038/s41598-021-93484-2)

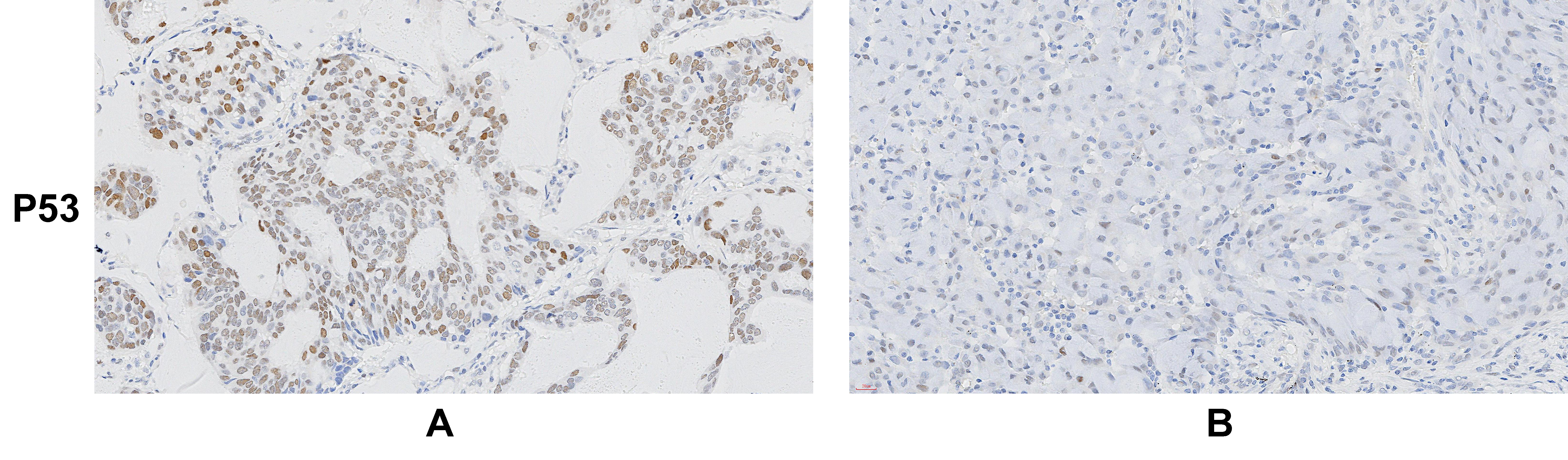

Supplement: Supplementary file 2 — Supplementary Fig. S1. [file 41598_2021_93484_MOESM2_ESM.tif]

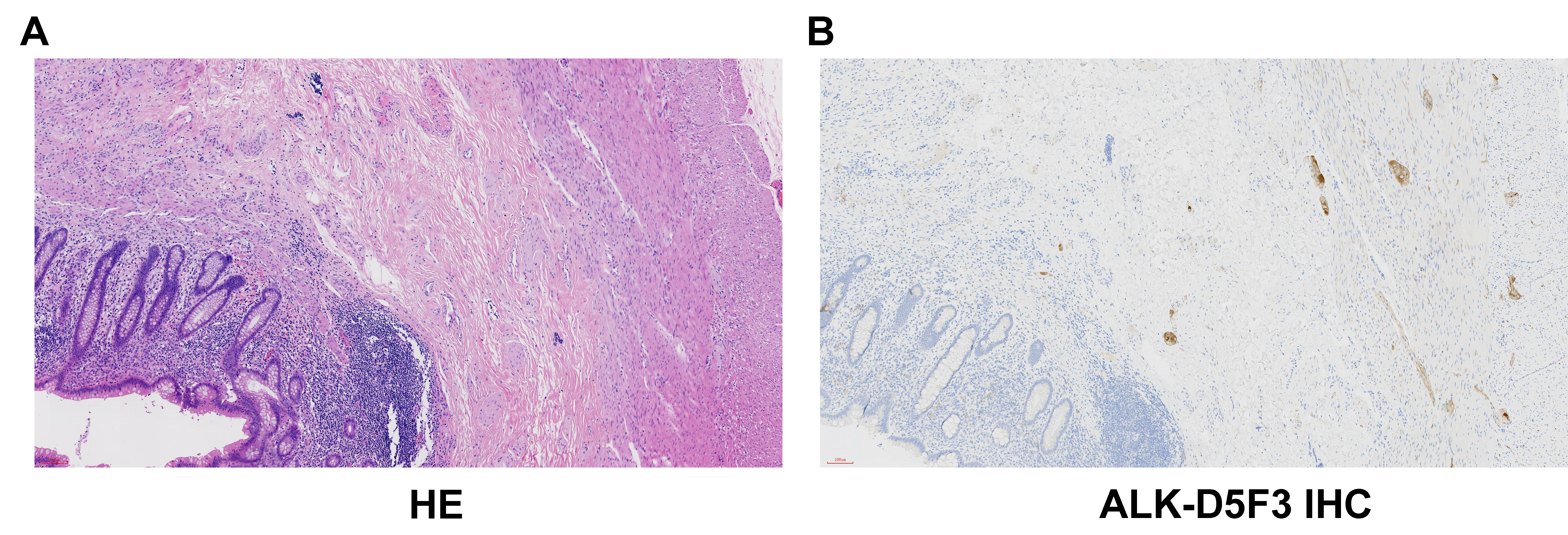

Supplement: Supplementary file 3 — Supplementary Fig. S2. [file 41598_2021_93484_MOESM3_ESM.tif]
