# Supplementary material for: SPP1 overexpression is associated with poor outcomes in ALK fusion lung cancer patients without receiving targeted therapy
Source: Sci Rep. 2021 Jul 7;11:14031. doi: 10.1038/s41598-021-93484-2 (PMC8263595; doi:10.1038/s41598-021-93484-2)
Supplement: Supplementary file 4 — Supplementary Table S1. [file 41598_2021_93484_MOESM4_ESM.docx]

Table S1: One ALK-positive lung cancer patient harbor pathogenic TP53 mutation.

| Hugo_  Symbol | Chromosome | Position | Ref_allele | Alt_  allele | Variant_  Classification | AAChange | Variant_Type | CGC | Comprehensive435 | SMG127 | Tumor_  Sample_Name | Gene_  Description |
| --- | --- | --- | --- | --- | --- | --- | --- | --- | --- | --- | --- | --- |
| TP53 | 17 | 7577126 | T | A | Missense_  Mutation | TP53:NM_001126115:exon4:c.A416T:p.E139V\|TP53:NM_001126116:exon4:c.A416T:p.E139V\|TP53:NM_001126117:exon4:c.A416T:p.E139V\|TP53:NM_001276697:exon4:c.A335T:p.E112V\|TP53:NM_001276698:exon4:c.A335T:p.E112V\|TP53:NM_001276699:exon4:c.A335T:p.E112V\|TP53:NM_001126118:exon7:c.A695T:p.E232V\|TP53:NM_000546:exon8:c.A812T:p.E271V\|TP53:NM_001126112:exon8:c.A812T:p.E271V\|TP53:NM_001126113:exon8:c.A812T:p.E271V\|TP53:NM_001126114:exon8:c.A812T:p.E271V\|TP53:NM_001276695:exon8:c.A695T:p.E232V\|TP53:NM_001276696:exon8:c.A695T:p.E232V\|TP53:NM_001276760:exon8:c.A695T:p.E232V\|TP53:NM_001276761:exon8:c.A695T:p.E232V | SNP | breast, sarcoma, adrenocortical carcinoma, glioma, multiple other tumour types | High Confidence Driver | 42.00% | PF_844110_9B | tumor protein p53 |
