# Supplementary material for: SPP1 overexpression is associated with poor outcomes in ALK fusion lung cancer patients without receiving targeted therapy
Source: Sci Rep. 2021 Jul 7;11:14031. doi: 10.1038/s41598-021-93484-2 (PMC8263595; doi:10.1038/s41598-021-93484-2)
Supplement: Supplementary file 5 — Supplementary Table S2. [file 41598_2021_93484_MOESM5_ESM.docx]

Table S2: TP53 gene counts of 5 ALK-positive lung cancer cases and the corresponding pericarcinous tissues by NanoString assay.

| Probe Name | Case 1 | Case 2 | Case 3 | Case 4 | Case 5 | Control 1 | Control 2 | Control 3 | Control 4 | Control 5 |
| --- | --- | --- | --- | --- | --- | --- | --- | --- | --- | --- |
|  | Solid(signet) | Solid(signet) | Micropapillary with mucin | Cribriform pattern of acinar with mucin, solid | Papillary,solid(signet),cribriform pattern of acinar with mucin |  |  |  |  |  |
| TP53 | 6 | 37 | 148 | 495 | 745 | 2 | 8 | 4 | 11 | 451 |
